# Face Biometric Spoof Detection Method Using a Remote Photoplethysmography Signal

**DOI:** 10.3390/s22083070

**Published:** 2022-04-16

**Authors:** Seung-Hyun Kim, Su-Min Jeon, Eui Chul Lee

**Affiliations:** 1 Department of AI & Informatics, Graduate School, Sangmyung University, Hongjimun 2-Gil 20, Jongno-Gu, Seoul 03016, Korea; 202134015@sangmyung.kr (S.-H.K.); 202132044@sangmyung.kr (S.-M.J.); 2 Department of Human-Centered AI, Sangmyung University, Hongjimun 2-Gil 20, Jongno-Gu, Seoul 03016, Korea

**Keywords:** convolutional neural network, face anti-spoofing, face recognition, long short-term memory, remote photoplethysmography

## Abstract

Spoofing attacks in face recognition systems are easy because faces are always exposed. Various remote photoplethysmography-based methods to detect face spoofing have been developed. However, they are vulnerable to replay attacks. In this study, we propose a remote photoplethysmography-based face recognition spoofing detection method that minimizes the susceptibility to certain database dependencies and high-quality replay attacks without additional devices. The proposed method has the following advantages. First, because only an RGB camera is used to detect spoofing attacks, the proposed method is highly usable in various mobile environments. Second, solutions are incorporated in the method to obviate new attack scenarios that have not been previously dealt with. In this study, we propose a remote photoplethysmography-based face recognition spoofing detection method that improves susceptibility to certain database dependencies and high-quality replay attack, which are the limitations of previous methods without additional devices. In the experiment, we also verified the cut-off attack scenario in the jaw and cheek area where the proposed method can be counter-attacked. By using the time series feature and the frequency feature of the remote photoplethysmography signal, it was confirmed that the accuracy of spoof detection was 99.7424%.

## 1. Introduction

In system security, there are three common categories for human authentication: “Something you know,” “Something you have,” and “Something you are.” “Something you know,” is the most common and well-known type of authentication. In this type of authentication, passwords are used for specific access. However, passwords are easily forgotten and can lead to serious problems, such as substantial losses [1]. “Something you have” can also be easily stolen by attackers or lost by the owner. Unlike the first two categories, “Something you are” cannot be as easily stolen by others or be forgotten or lost because it is an authentication based on what the owner looks or sounds like (i.e., biometric authentication). The most common methods based on this kind of authentication are iris scans, fingerprint readers, voiceprints, and face readers. As these are already part of our daily lives, it is easy to think of examples, such as smartphone authentication, Siri in iOS devices, Bixby in Samsung devices, and Smart Pay and other in-app payment systems. Because of its convenience, increasingly more applications tend to use biometric authentication.

However, there are still some challenges to overcome with biometric authentication. For example, voice recognition cannot be used in noisy situations and places that are too quiet, such as a library. Further, iris scanners can have security issues, as in the case of the Samsung S8 phone [2]. In addition, even the well-known fingerprint biometric recognition system had a security problem on the Samsung S10 smartphone [3]. Therefore, we believe that face authentication based on ID is the most promising type of security system because it is very user-friendly and can be easily applied to the device, requiring only an RGB camera. Moreover, with the increasing numbers of contactless and fully automated service systems in more fields owing to COVID-19, the number of areas in which facial recognition authentication is being applied is also increasing [4]. However, the existing face recognition technology is sensitive to changes in lighting or face angle, and several spoofing attacks are also possible for systems that use face recognition [5]. Consequently, research geared towards solving this problem is actively underway [6].

Photoplethysmography (PPG) is a simple optical technique for detecting blood volume changes in the microvascular bed of tissues [7]. More recently, a simpler method called remote PPG (rPPG) detection has been used and studied in many fields. The advantages of rPPG are that it can be extracted with RGB cameras without the need for additional equipment to detect existing PPGs and it is non-contact. Thus, people can use it with confidence in pandemic situations such as COVID-19. There are several uses for rPPG, and our laboratory has conducted several rPPG studies [8,9,10,11,12]. In one of the studies conducted, rPPG signals were used for face anti-spoofing [12]. Our research has since advanced even further based on the assets of the laboratory.

According to Ming et al. [13], face spoofing detection using rPPG signals has a significant advantage over 3D mask attack scenarios compared to other detection methods, such as 3D geometry cue-based methods or texture cue-based methods. For this reason, previous studies [14,15] that focused on face anti-spoofing based on rPPG have highlighted the good performance on 3D masks as a strength or combined it with other mechanisms [16]. However, using the rPPG signal alone can be a great advantage in implementations on mobile devices because only the RGB camera is needed to extract the signal. Nevertheless, when using the rPPG signal by itself, detecting replay attacks can be difficult because high-quality video can mimic the rPPG signal as if it were real facial data. Consequently, the rPPG signal is often used in combination with 3D geometric data, which can negate the advantage of ease of implementation. Therefore, our study focused on improving the accuracy of replay attack detection using the rPPG signal alone.

In previous work [12,17], frequency domain data have been extracted from the rPPG signal. However, in this study, we retained the sequential characteristics of the signal data to train deep learning models. The reason is that we surmised that data loss occurring during the conversion process of signal data to frequency domain data would affect the detection of replay attacks. In addition, in previous studies [17], additional processing such as comparison with components of the background area other than the face area is required. This can result in moving objects in the background causing interference in the process. Therefore, in our study, we avoid these risk factors by extracting signals only from the face region. Additionally, we devised several other spoofing attack scenarios to account for exceptions. Along with the well-known attack scenarios, such as printed photo attacks and replay video attacks used in previous studies [18], we wanted to detect attacks, such as photos with a part of the face cut out, and replay videos with light movements or microscopic vibrations. These attacks could be performed by attackers who already know that the forehead and cheeks have dense capillary vessels—and can therefore provide stronger and clearer rPPG signals than other areas [19]—and that a vibrating photo or shaking the light shining on the facial area can mimic the pixel changes of the rPPG signal.

## 2. Materials and Methods

**Figure 1:**
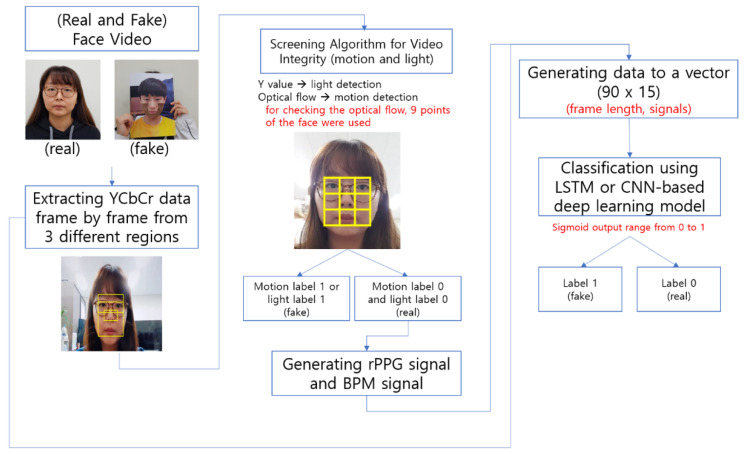
Overall process of our proposed algorithm.

The process flow of our proposed method for detecting face spoofing is shown in Figure 1. The input data are based on 3 s of image information, and the instrument used for data acquisition is an RGB camera. First, the face area is detected and captured by the RGB camera. The actual face area used is a composite of three regions: (a) the entire face area, (b) the area around the nose, and (c) the area around the eyes. This differs from the regions of interest used in previous studies [17,19]. We chose these different setting areas because they are difficult to conceal with hair or a mask. Furthermore, by focusing on those areas, we can effectively detect attack scenarios in which a photo with cut cheeks and chin is used. We surmised that such an attack scenario can be devised by an attacker who is familiar with rPPG-based face anti-spoofing. Subsequently, the color information extracted from the corresponding focus areas is converted into the YCbCr color model. This separates the brightness value and the color information. As stated by Phung et al. [20], the YCbCr color model can use other skin color information besides skin brightness depending on the race. Therefore, it is widely used to detect changes more effectively in human skin color information and blood flow expressed in the skin [12]. Then, an algorithm is applied to screen the extracted signal information. A detailed description of the screening algorithm for video integrity procedure is given in Section 2.2. Subsequently, images that are not detected as an attack are detected using a deep learning model. The signals used to train the model are explained in more detail in Section 2.3 and the models used are explained in Section 2.4.

### 2.1. Dataset

The data used in this study were collected with a total of two devices. In February 2022, Android-based and iOS-based mobile device users constituted 70% and 28% of mobile device users worldwide, respectively [21]. This means that the data in the mobile environment are used by a total of 98% of people. Owing to the characteristics of the rPPG signal, we acquired the video in an environment of 300–600 lux, which is typical indoor brightness without any separate lighting device. As each experimenter acquired data in a variety of spaces rather than a limited space, it was possible to obtain data in an environment with variations in brightness.

All subjects gave their informed consent for inclusion before participation. The study was conducted in accordance with the Declaration of Helsinki, and the protocol was approved by the ethics committee of Sangmyung University Institutional Review Board (IRB-SMU-S-2021-1-005). Based on the 13-1-3 system of the Enforcement Regulations of the Act on Bioethics and Safety of the Republic of Korea, ethical review and approval were waived (IRB-SMU-S-2021-1-005) for this study by Sangmyung University Institutional Review Board, because this study uses only simple contact measuring equipment or observation equipment, without any physical changes.

#### 2.1.1. Devices

Two devices were used to obtain the video data: a Samsung Galaxy S8 (Android-based OS) and an iPad Air 4th generation (iOS). Both devices used rear-facing cameras, the image quality was FHD, and the size of the captured image was 1920 × 1080. In addition, the video was filmed at 30 fps.

#### 2.1.2. Dataset for Screening Algorithm to Confirm Video Integrity

We did not use open data for face anti-spoofing, such as the existing open CASIA-FASD, CelebA-Spoof, or MSU-MFSD. In such datasets, most of the motions that are unnecessary for classification in the static state that we are targeting are detected, resulting in the real data that should be passed also not passing the screening algorithm for video integrity. In addition, because there is no video of the attack in which the attack scenario we want to detect is implemented, we thought that the model could learn unnecessary factors, such as image quality, frames per second (fps), and human characteristics, if only the comparison group data are used and only the attack data are recreated.

**Figure 2:**
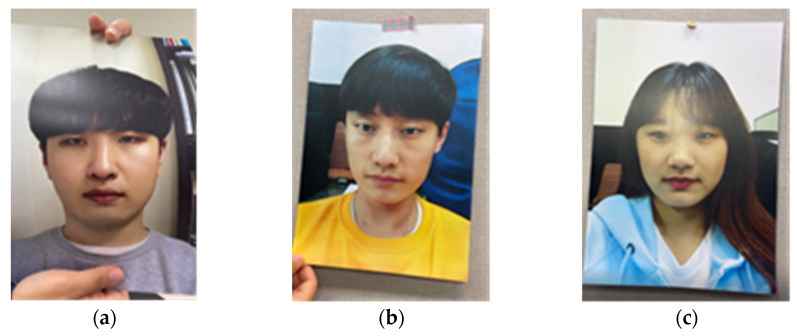
Sample captured images of data for the screening algorithm for video integrity: (**a**) bending photo attack; (**b**) slight shaking attack; (**c**) light movement attack.

Therefore, data collection was performed separately for the two situations. First, images of attack scenarios were collected for detection by the screening algorithm for video integrity before model learning, and then the data that passed the screening algorithm were collected. Video data were collected for a total of four different attack scenarios and filmed in three different environments. The attack scenarios to be detected in our screening algorithm for video integrity were as follows: (1) slight shaking, (2) bending the photo horizontally, (3) bending the photo vertically, and (4) light moving in the face region attacks. Figure 2 shows a sample image for each case. In the case of the rPPG signals detected in these attacks, unnecessary tendencies may be learned if classification learning is performed on the model because they tend to mimic changes in the pixels caused by changes in human blood flow. Therefore, we used the screening algorithm for video integrity to detect the attacker when unnecessary movements and changes occur. Classification using the deep learning model was performed on videos that passed the screening algorithm with signal stage.

#### 2.1.3. Dataset for Model Training

We collected data that did not cause unnecessary movement, except for biological signals (change in blood flow, movement due to breathing, blinking, etc.). First, we collected videos of static images of real people for the comparison group. In our experiment, 18 people participated and the images were recorded for approximately 1 min, which is long enough to proceed with data augmentation later. To prevent the model from learning unnecessary elements, factors other than the targeted classification criteria (real face or attack) were controlled during acquisition.

Below is a video of two attack scenarios adopted in the experiment. The first attack is a high-quality replay attack, which is known to be difficult to detect using biosignals [13,16]. We replayed the video recorded on the Android and iOS devices, respectively, and re-recorded each other’s videos. In the next attack, the face image is cropped and further processed. We chose two methods for cropping. In the first method, the cropped photo of the eyes, nose, and mouth were placed on the face. This is a method of active recognition rather than passive recognition in existing studies. It is widely used in approaches where the attack detector detects spoofing by identifying liveness through movements, such as blinking [22,23] or head movements [24,25,26,27].

**Figure 3:**
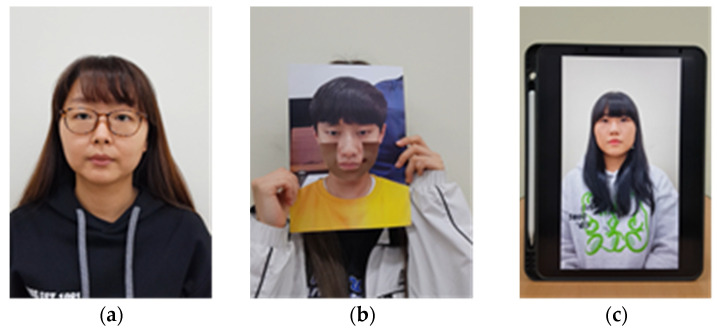
Data used for model training: (**a**) real face data; (**b**) cut off photo attack example; (**c**) replay attack example.

Subsequently, we performed an attack using our assumed characteristics of rPPG. It is well-known that rPPG data are well detected in the cheek and forehead regions [17]. Therefore, rPPG data are typically collected from the cheeks or forehead [19]. Because the influence of this area is large, attackers can develop an attack method that conceals the cheek and chin area, rather than the eyes, nose, and mouth. An example is shown in Figure 3. When the cheek and chin areas are cut, changes in blood flow are detected by the camera and rPPG and beats per minute (BPM) data are output in a form similar to that of a real person. Figure 3 shows an example image for each video type.

#### 2.1.4. Data Augmentation

We collected data from six cases. Eighteen videos were collected for each case, approximately 1 min and 10 s in length, and collected at 30 fps. Of the 18 videos for each case, 15 were used for training and three for testing. Thus, 18 of the 108 videos were used for testing and 90 were used for learning, a distribution of 5:1. Then, the videos were divided into clips for augmentation. Each clip is 90 frames in length, and each clip was acquired using a sliding window method of 10 frames. The total number of clips used for learning and testing is shown in Table 1.

### 2.2. Screening Algorithm for Video Integrity

As mentioned in Section 2.1.2, when using rPPG signals to detect spoofing attacks, some attack scenarios can be detected in advance, such as motion attacks and light movement attacks. Owing to the characteristics of rPPG data extraction, the smallest movements are considered liveness movements. Therefore, we detected these types of attacks in advance to increase accuracy.

Different algorithms were used for motion and light changes. In the case of motion, the change distance of each point for each frame was used as the value using optical flow, and in the case of light, the Y value from the previously extracted YCbCr data was used.

**Figure 4:**
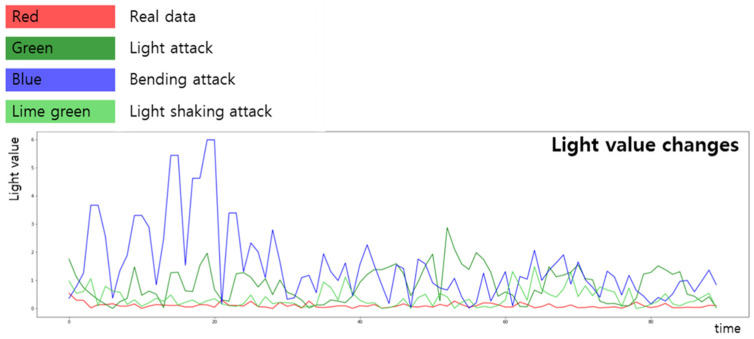
Light value changes according to time domain.

Figure 4 presents a graph showing the change in the light value over time. Over time, the previously extracted light value of the actual face changes little. By contrast, the light values in the three attack scenarios change substantially.

**Figure 5:**
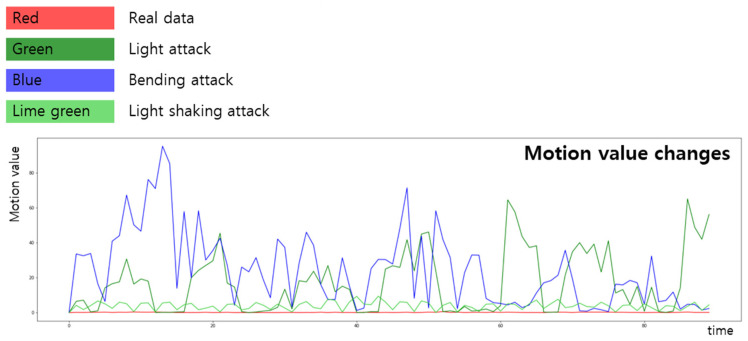
Motion value changes according to time domain.

Figure 5 is a graph showing the nine distance changes set as points in the optical flow [28] used as the value of movement. As can be seen in the graph, it has been confirmed that the three attack scenarios change significantly over time, whereas the actual facial data show little movement.

**Figure 6:**
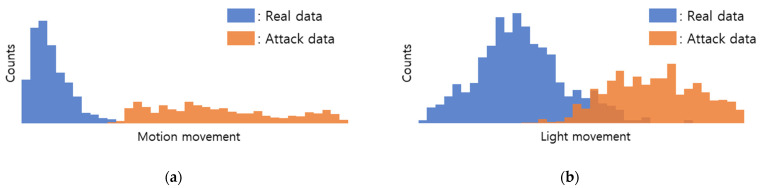
Genuine/Imposter plot of (**a**) motion and (**b**) light movement.

Therefore, we applied a screening algorithm that detects attacks based on motion and light changes based on the magnitude of the change. The upper plot in Figure 6 is the average distribution of the motion value by video, and the lower plot is the average distribution of the Y value, which is the light detection value. Blue is the average value of data without attack, and red is the average value distribution of the data with motion attack. As can be seen, most videos show that the value is shifted to a small value when there is no attack.

One of the datasets presented in Section 2.1.2 was used to evaluate these screening algorithms for video integrity. Attacks were detected 100% of the time and only videos without unnecessary movements were passed to the next step.

### 2.3. Signal Extraction

**Figure 7:**
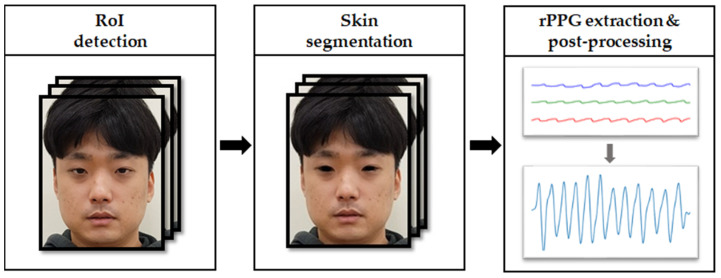
Overview of the propose rPPG signal extraction.

Five different signals were used to train the model. First, the most key signal in our research is the rPPG signal. The method of extracting rPPG uses the algorithm made in our lab. A brief description of the algorithm is shown in Figure 7 and a more specific description of the algorithm is provided as follows.

First, we used the face detector on the OpenCV DNN module [29] to detect the front face of the first frame. Then, the kernelized correlation filter tracker was used to track the detected faces in subsequent frames. This step allows you to position your face reliably while minimizing background pixels. Because there is no pulse information in the background, a statistical filtering method based on the YCbCr color space is applied to filter background pixels from the face rectangle. The YCbCr variant of the RGB color space is shown in Equation (1).

(1) $$Y^{\prime }=16+\left(65.481\cdot R^{\prime }+128.553\cdot G^{\prime }+24.966\cdot B^{\prime }\right)\;C_{B}=128+\left(-37.797\cdot R^{\prime }+74.203\cdot G^{\prime }+112.0\cdot B^{\prime }\right)\;C_{R}=128+\left(112.0\cdot R^{\prime }+93.786\cdot G^{\prime }+18.214\cdot B^{\prime }\right)$$

Here, the heuristic method was used to determine the extent of the skin. Static threshold ranges of 133 ≤ *C_B_* ≤ 173 and 77 ≤ *C_R_* ≤ 127 were used [30]. The CHROM (chrominance) method is then used to extract the rPPG. CHROM algorithms are often used for rPPG extraction because they are strong in subject motion. Because the signal still contains noise components, two post-processing steps were applied to further improve the signal quality. First, remove the trend such as breathing from the signal, and then apply the detrending to obtain a zero-center normalized signal. It also applies Butterworth band pass filtering at cutoff frequencies of 42 and 240 bpm to remove components unrelated to heart activity. The general formula for the Butterworth bandpass filter is as shown in Equation (2). The overall process is much the same as in [31].

(2) $${\left|H_{a}\left(jΩ\right)\right|}^{2}=1/\left\{1+{\left(\frac{Ω}{{Ω}_{c}}\right)}^{2N}\right\}$$

In addition to rPPG, BPM data and the raw signal from YCbCr were also used (YCbCr data were decomposed into three different signals: *Y*, *C_B_*, and *C_R_*). Fourier spectral method in Equation (3) was used to generate BPM data.

(3) $$f\left(t\right)=\frac{1}{2\pi }\int_{-\infty }^{\infty }F(\omega )e^{i\omega t}d\omega$$

It helps to remove noise from the signal that is not related to the pulse. We also apply the Hann Window prior from [31] to mitigate spectral leakage. Lastly, the power spectral density (PSD) in Equation (4) was used to detect the maximum power peak and multiplied 60 to estimate the pulse rate in BPM.

(4) $$PSD\left(k\right)=\frac{2\;di}{n^{2}}\left({\left(Y_{real}\left(k\right)\right)}^{2}+{\left(Y_{imag}\left(k\right)\right)}^{2}\right)$$

The signals were extracted from three different face regions: the face as a whole, the nose region, and the eye region. As mentioned earlier, using different regions has an advantage in detecting the cropped photo attack.

### 2.4. Model

#### 2.4.1. LSTM-Based Model

**Figure 8:**
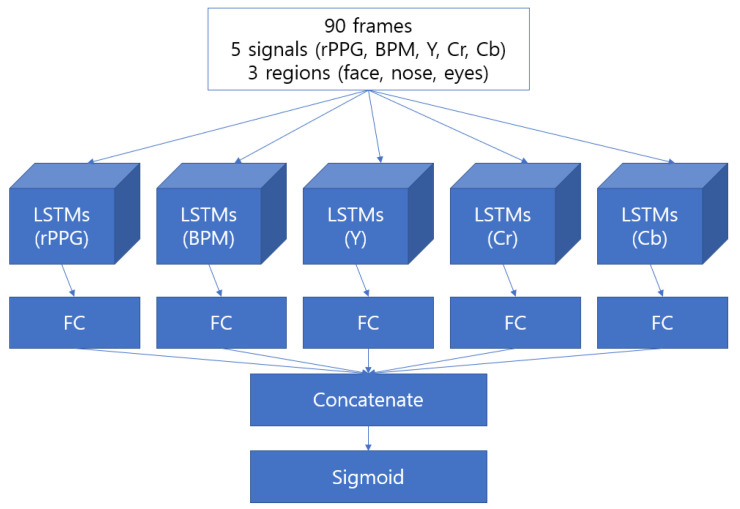
LSTM-based model structure with every method implemented.

As the signal data are sequential, a long short-term memory (LSTM) model was used to train the signal data. Our initial goal is to learn rPPG and related signals individually and derive results from them. Therefore, we selected and used recurrent neural network (RNN) type models. Figure 8 shows a brief structure of the model. The number of layers of the model was increased from one to three. As more than three layers did not provide much performance improvement, three layers were used as the structure. Additionally, in the case of a dropout, after the last fully connected layer, we went to a size of 0.5 to avoid overfitting the model.

#### 2.4.2. Convolutional Neural Network-Based Model

The convolutional neural network (CNN)-based model is based on the concept that nearby signal changes may be more relevant than time-series features in detecting face spoofing. Existing studies have shown that face spoofing by rPPG uses more frequency-based information derived by Fourier transform than the overall signal flow. It can be seen that this focuses more on the frequency change of the partially generated signal than on the sequential characteristics of the signal. Therefore, we also developed a CNN-based model that uses CNN-series models to learn the degree of change in nearby signals.

**Figure 9:**
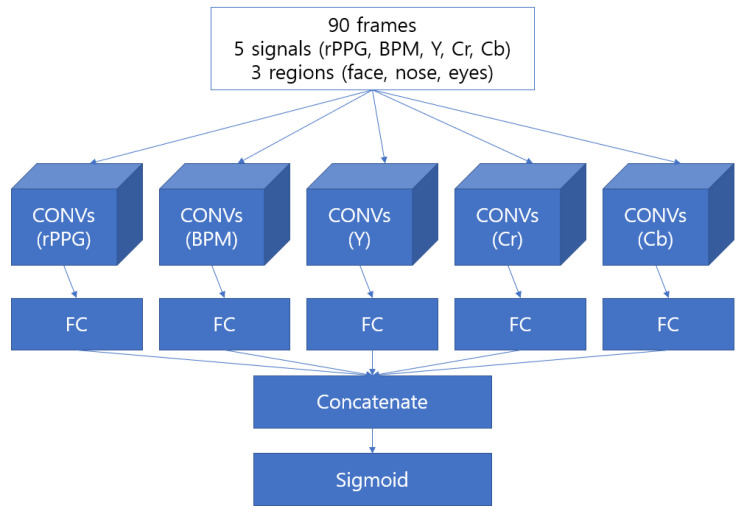
Structure of our CNN-based model with every method implemented.

CNN models currently use very deep-layer models such as ResNet and InceptionNet for learning. However, the size of our data is (90, 15), which is much smaller than the size of the commonly used (112, 112, 3) or (225, 225, 3). Therefore, we surmised that learning would not progress if the number of layers of the CNN was too deep, and five layers of AlexNet [32], an early CNN-like network, were used. Furthermore, additional layers were also used to avoid overfitting, such as batch normalization and dropout, based on AlexNet. Our final CNN-based network differed from the existing frequency-based detection method in that it not only learns the characteristics of frequency, but also learns the sequential feature by repeating the process of compressing, collecting, and compressing the features of nearby signals and leaving them in a sequential manner. Figure 9 shows the structure of the model.

#### 2.4.3. CNN with LSTM Model

**Figure 10:**
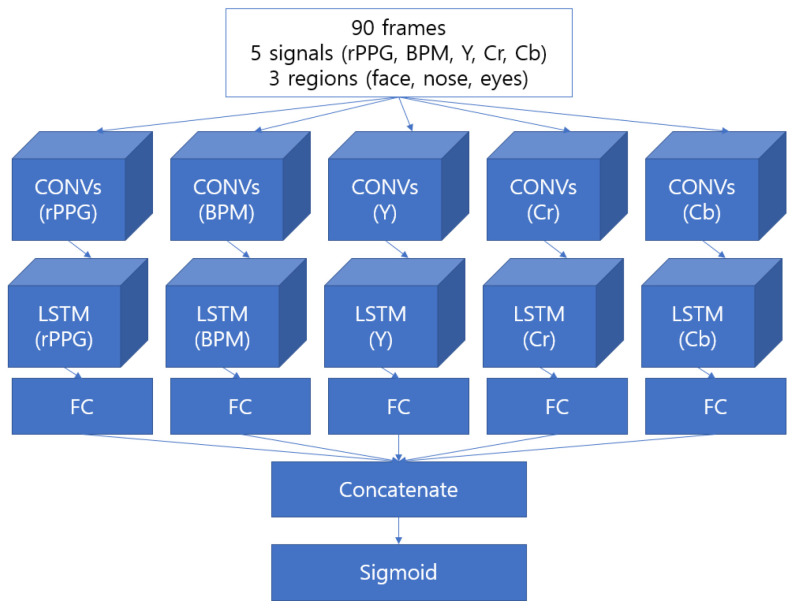
Structure of our CRNN-based model with every method implemented.

The following model is a Convolutional Recurrent Neural Network (CRNN) that learns the sequential information of the signal through LSTM, a model of the RNN series, after learning the information of the nearby signal through CNN. The CRNN model has been used to recognize image-based sequential data [33]. After extracting the features of partial images through the feature map using the CNN, the corresponding feature map is converted into a feature sequence and used as input to the RNN. Figure 10 shows the structure of the model.

## 3. Results

The results for the model were evaluated via two metrics: accuracy and area under the curve (AUC). Accuracy indicates how well the test clips that are not used in the training set are evaluated. It is calculated as the number of correct clips over the total number of test clips. AUC signifies the area below the receiver operating characteristic curve (ROC) [34]: the closer it is to one, the better is the performance. This indicator has the advantage of being able to evaluate sensitivity and specificity in combination. The best accuracy was obtained when three different regions of the face were used, and five signals were trained separately. Table 2 shows that the performance is higher when extracted from three areas than when extracted only from the face. Further, the performance is better when combined in the last floor after learning each of the five signals through separate layers. In addition, we confirmed that the data used have a sequential characteristic as signal data but perform slightly better when using a CNN-based network model than when using an RNN-based model, LSTM.

The signal data we use as input confirmed that the network with the CNN model has slightly higher performance than the network with the RNN series, but we still conducted an experiment to learn our final input data with the CRNN model because learning the sequential features of the signal in RNN models is more suitable.

As can be seen from the results in Table 3, we confirmed that the CRNN model suite does not significantly improve performance when only CNN or RNN models are used.

We also extracted the accuracy for each case. Table 4 shows that most cases were accurately predicted, and that iOS-based devices have slight errors. It can be seen that there is a difference in the signal of the input by device.

## 4. Discussion

In this study, we focused on retaining the sequential property rather than using the frequency value from Fourier transform. We also aimed to identify attacks that are considered difficult for rPPG. High-quality replay attacks are commonly considered vulnerable to traditional rPPG-based anti-spoofing models that use only rPPG. This is because high-resolution images can be displayed by imaging changes in the human blood flow. Therefore, it is necessary to use additional cameras that measure depth or actively perform certain actions (e.g., blinking or head movements). However, such systems are difficult to use in mobile environments or are inconvenient for users.

**Figure 11:**
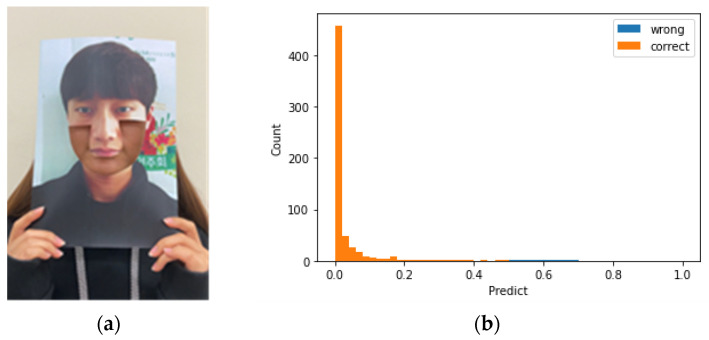
(**a**) Error case when fake was predicted as real. (**b**) Genuine and imposter plots for real test data in iPad mini case.

We therefore investigated whether the rPPG signal by itself can distinguish replay attacks and used YCbCr data that can be collected together when extracting rPPG and BPM data that can be extracted later to obtain an accuracy of 99.7472% and an AUC of 0.9997 for CNN-based signal models. There was one case where a fake video was predicted as real, when the cheek and chin area in the photo was exposed, as shown on the left in Figure 11. This is an attack scenario we presented where a large portion of the face was exposed. Out of 198 clips, only three clips from the video were found to be genuine. Even for the case where a real face was detected as fake, the result was not greater than 0.65, which is close to the threshold shown on the right in Figure 11. In addition, in the experiment, a proposed attack with a photo that had a large portion of the face (from cheek to chin) cut out where rPPG can be detected, rather than an attack of a previously well-recognized printed photo, was also confirmed with virtually 100% accuracy. Compared to the results reported by Suh and Lee [11] using only rPPG signals from RGB cameras, we confirmed that the performance of our proposed method was better in the high-quality replay attack scenario. According to the results of the experiment we reproduced with our dataset, Table 2 shows that the performance of [11] is 69.5964% for the LSTM based model and 73.7550% for the CNN based model.

## 5. Conclusions

Facial recognition technology is a highly promising type of security system because it is user-friendly and can be easily applied to devices. The algorithm proposed in this paper not only removes the sensitivity to changes in lighting or face angle, which is a weakness of existing face recognition technologies, but is also robust against facial movements and minute tremors. It also minimizes the vulnerability to certain database dependencies and high-quality replay attacks without additional devices. As facial recognition technology is increasingly being used in the mobile realm, we expect to see more and diverse types of facial spoofing attacks in the future. We also believe that new attackers will emerge with technical knowledge of face spoofing anti-spoofing techniques. This study shows a new research direction and potential for expansion in the field of face anti-spoofing. We anticipate that this study will enable many types of face spoofing detection data to be created in more diverse environments, and detection algorithms to be prepared not only for existing attacks, but also for various scenarios, so that users can use face recognition conveniently and safely in mobile environments. In future research, we will determine how to acquire more data and videos from different environments and detect these different attacks using rPPG data and deep learning models.

## Figures and Tables

**Table 1:** Number of clips for each case.

Device	Video Type	Train	Test	Total
Samsung S8	Real	2974	592	3566
	Cut off photo attack	2971	595	3566
	Replay attack	2775	554	3329
iPad Air 4th	Real	2986	606	3592
	Cut off photo attack	2990	598	3588
	Replay attack	2770	549	3319
Total		17466	3494	20960

**Table 2:** Accuracy and AUC of the LSTM- and CNN-based models.

	LSTM-Based		CNN-Based	
	Accuracy (%)	AUC	Accuracy (%)	AUC
Face only	69.5764	0.736579	73.7550	0.766207
3 regions	94.9341	0.976318	93.8465	0.917558
3 Regions/5 signals trained separately	98.7979	0.997034	99.7424	0.999736

**Table 3:** Accuracy and AUC of CRNN-based model.

	CRNN-Based	
	Accuracy (%)	AUC
3 Regions/5 signals trained separately	97.6817	0.996146

**Table 4:** Accuracy of each case with LSTM, CNN, and CRNN models (%).

	LSTM	CNN	CRNN
Real data (Galaxy S8)	100.0	100.0	100.0
Real data (iPad Air)	93.0693	99.5049	91.2541
Cut photo attack (Galaxy S8)	100.0	100.0	100.0
Cut photo attack (iPad Air)	100.0	98.99665	95.3177
High-quality replay attack (Galaxy S8)	100.0	100.0	100.0
High-quality replay attack (iPad Air)	100.0	100.0	100.0

## Data Availability

The obtained data cannot be shared because it was agreed that it could be used only for this study.
